# The Study of Walking, Walkability and Wellbeing in Immersive Virtual Environments

**DOI:** 10.3390/ijerph18020364

**Published:** 2021-01-06

**Authors:** Amit Birenboim, Pazit Ben-Nun Bloom, Hila Levit, Itzhak Omer

**Affiliations:** 1 Department of Geography and the Human Environment, Tel Aviv University, Tel Aviv 6997801, Israel; hilalevit@mail.tau.ac.il (H.L.); omery@tauex.tau.ac.il (I.O.); 2 Department of Political Science, The Hebrew University of Jerusalem, Jerusalem 9190401, Israel; pazit.bennun@mail.huji.ac.il

**Keywords:** walkability, immersive virtual environment, VR, walking simulator, wellbeing, eye tracking, gait analysis, electrodermal activity, heart rate, mobility

## Abstract

Recent approaches in the research on walkable environments and wellbeing go beyond correlational analysis to consider the specific characteristics of individuals and their interaction with the immediate environment. Accordingly, a need has been accentuated for new human-centered methods to improve our understanding of the mechanisms underlying environmental effects on walking and consequently on wellbeing. Immersive virtual environments (IVEs) were suggested as a potential method that can advance this type of research as they offer a unique combination between controlled experimental environments that allow drawing causal conclusions and a high level of environmental realism that supports ecological validity. The current study pilot tested a walking simulator with additional sensor technologies, including biosensors, eye tracking and gait sensors. Results found IVEs to facilitate extremely high tempo-spatial-resolution measurement of physical walking parameters (e.g., speed, number of gaits) along with walking experience and wellbeing (e.g., electrodermal activity, heartrate). This level of resolution is useful in linking specific environmental stimuli to the psychophysiological and behavioral reactions, which cannot be obtained in real-world and self-report research designs. A set of guidelines for implementing IVE technology for research is suggested in order to standardize its use and allow new researchers to engage with this emerging field of research.

## 1. Introduction

The beneficial outcomes of walking activity have been increasingly affirmed during the last two decades [1,2,3]. Walking is considered a sustainable low-carbon form of mobility [4,5], an active mode of transportation that contributes both to the physical and mental health of individuals [6,7,8,9] and to a just mode of transportation that may support greater social equality due to its affordability [10]. Furthermore, walking is associated with facilitating social capital and a sense of community as well as stimulating social interaction and an improved sense of security [11,12,13,14,15], all of which support city livability and better performance of urban areas [16,17,18]. Being a healthy, sustainable and just activity and having a positive impact on urban living, walking coincides with recent public policy trends that endeavor to promote wellbeing [1,19]. In this regard, walking has been associated both with “eudemonic” aspects of wellbeing, as a result of its effect on the maximization of one’s virtue, autonomy, and social interactions, and “hedonic” aspects of increasing pleasure as a result of the positive feelings and affects associated with it [20].

Urban environments in particular seem to have received much attention in regard to walking policy as the implementation of walking promotion interventions is commonly more efficient in denser urban areas where land-use mixture and geographical proximity between people allow city dwellers to engage in many types of activities within walking distance of their homes [21,22]. With the growing awareness of walking’s potential for improving wellbeing in mind, many efforts are now being directed to investigating factors that make environments more pedestrian-friendly and that will consequently increase walking activity in cities [17,19,22,23,24]. A concept that became central in this regard is *walkability*, which “represents the extent to which the built environment facilitates or hinders walking for purposes of daily living” [2] (p. 1926). It is commonly used to describe how varied characteristics of the built environment influence the frequency and quality of walking [25].

There are substantial indications that walkable environments have a positive effect on residents’ health and wellbeing [7,21,24]. Residents of more walkable neighborhoods experienced a smaller increase in weight, systolic blood pressure, and high-density lipoprotein cholesterol [26] and also reduced overweight and obesity as well as associated problems including incidence of diabetes [26,27]. Walkable environments were also found to support social interactions and the human need for simulation [20,24,28] and even to increase creativity [29].

Neighborhoods’ physical, environmental, and social characteristics, constructed by human activity, shape their walkability and therefore affect residents’ walking frequency and durations [17,23]. Among these characteristics are ones shaped by urban planning policies, such as traffic speed and density [19,30], traffic safety [30,31,32], street connectivity [19,31], residential density [19,30,31], and land-use mix, which allows accessibility to varied local businesses (e.g., restaurants, food stores, retail) or other public services [3,30,33]. The perceived attractiveness of neighborhoods and pedestrian-friendly design are also positively associated with high walking frequency. These include, among other things, well-provisioned sidewalks [32,34], well-maintained lighting [32], greenery [35], cleanliness [34,36], and shaded streets [36].

Much of the research on walkability and wellbeing in both the health and transportation-planning disciplines has relied on more classical transportation approaches that examine the correlation between environmental and contextual characteristics (often of the home neighborhood) to the health and wellbeing of residents or to walking durations, distances and frequencies [17,21,23,34,37,38]. In recent years, researchers show more interest in human-centered approaches that focus on experiential and affective dimensions of walking [1,2,20,39,40,41] and especially on how the experience of walking environments may affect walking behavior [22]. For this purpose, more fine-grained approaches and methods that go beyond census-like surveys and which consider the specific characteristics of individuals and their interaction with the immediate environment (i.e., the environment that they can experience through their senses) are adopted [2,19,22,40].

In the current article, we review, test and evaluate the feasibility of employing a novel immersive virtual environment (IVE) technology for studying walking behavior, which may further support the investigation of experiential and affective dimensions of walking that are associated with wellbeing. IVEs are both highly realistic and controlled. This unique combination allows performing lab-style studies that enjoy high internal validity due to their experimental design, on one hand, and enhanced ecological validity due to their mundane realism, on the other. Furthermore, IVEs make it possible to implement advanced behavioral and physiological measurements that go beyond self-reporting. These measurements can be collected in extremely high resolutions of parts of meters and seconds, resolutions that are difficult to impossible to use when using more traditional methods. Hence, IVEs may support a detailed analysis of objective walking parameters (e.g., by extracting speed, acceleration and gait characteristics), wellbeing (e.g., using biosensors that serve as markers of emotional arousal and stress) and the relation of the two to the immediate environment (e.g., through accurate recording of participants’ locations in the IVE and using eye-tracking monitoring). This type of measurement has great potential to enhance the study and planning of the walkable environment.

## 2. Using Virtual Reality to Study Human Behavior and Mobility

IVE technology has been available for several decades and has been utilized for research purposes since at least the 1990s [42,43]. Two main types of IVE technologies have been developed and used over the years. A *cave automatic virtual environment* (CAVE) utilizes a set of screens, projectors and motion-detection cameras within a designated cubic room. In *head-mounted display* (HMD) technology, visual content is observed through small display optics that are affixed to a headset and which is equipped with a set of inertial and other sensors that track the headset and user’s movement and position. Each of the technologies has its own advantages and drawbacks. CAVE, for example, supplies a superior field of view and allows users to observe their own body while engaged with the IVE, which generates a more natural experience. HMD, on the other hand, supplies a more immersive experience as it isolates users from the external environment. It also requires a smaller physical space for its operation than does CAVE (for a more comprehensive comparison between the technologies see, for example, [44]). While IVE technology has been available for some time now, it is only with the recent introduction of relatively inexpensive HMD headsets (Oculus Rift and HTC Vive released a consumer version in 2016 and development kits were available even earlier) that IVE technology is beginning to be more widely adopted for commercial and research purposes. To generate a more dynamic and interactive experience, various types of virtual reality (VR) input devices have been developed [45], of which the most common are hand controllers that allow basic interaction of users with the IVE through their hands (e.g., by moving the hand and/or pressing buttons). A few VR locomotion devices that allow omnidirectional walking for unlimited distances (independent of room size) have also been recently introduced [46,47]. Here, we test and evaluate the utilization of these newly available devices for studying walking behavior. Until now, studies that investigated walking behavior in virtual environments utilized less realistic movement controllers such as hand controllers and joysticks (see, for example, [47]), which, as will be explained in the following paragraphs, has a number of limitations. 

What is often considered the most prominent advantage of IVEs in the context of human behavior research is that they allow researchers to generate highly realistic and complex environments, while maintaining full control over participants’ exposure to environmental elements and stimuli [43,48]. This unique combination may help researchers confront the long-standing dilemma of choosing between experimental control and ecological validity [42,48,49]. In other words, IVEs allow researchers to perform lab-style controlled experiments that support inference of causality, without compromising on the realism that is essential for translating the results into valid insights relevant to real-world situations. However, IVEs differ in their level of realism and in cases where users conceive an IVE to be inconsistent with real environments, the (ecological) validity and quality of the study are jeopardized [50].

Immersion and presence are the two concepts most commonly used to evaluate IVEs’ realism level. Immersion relates to the objective properties of a VR system and the “extent to which the computer displays are capable of delivering an inclusive, extensive, surrounding and vivid illusion of reality to the senses of a human participant” [51] (p. 606). Presence relates to the subjective aspects of users’ perceptions of the simulated environment and more particularly to the degree to which users feel as if they are present in the displayed environment even though they are not physically there [51,52]. The two concepts are related to one another in the sense that the higher the immersion level of an environment, the greater the participants’ sense of presence is [53,54]. It is assumed that when the displayed environment has a high level of (immersion and) presence, participants’ behavior will be more consistent with daily, real-world behavior [48,51] and hence the study will enjoy greater ecological validity. Sense of presence has been a central subject of inquiry within VR research and several methods and questionnaires have been developed to assess it [55,56].

Until now, only a handful of studies have tested the quality of walking experience using IVEs. Using quantitative questionnaires and semi-structured interviews, Boletsis and Cedergren [57] compared user experiences of three common locomotion IVE techniques: (1) walking-in-place (without an omnidirectional treadmill); (2) controller/joystick-driven movement, where participants use a hand controller or joystick to direct their movement; and (3) teleportation, in which users advance from one teleportation point to another by pointing with the hand controller to the next point (i.e., advancement in the IVE is made through non-continuous jumps). The researchers concluded that the walking-in-place technique generated a greater sense of immersiveness due to the naturalness of the walking, as well as enjoyment. However, walking-in-place scored worst in the ease-of-use dimension and generated more side effects, such as tiredness and psychophysical discomfort (due to fear of collision and motion sickness). Teleportation that relies on non-continuous movement was rated at the lower end in many of the dimensions examined. While Boletsis and Cedergren did not use omnidirectional treadmill hardware, their study implies that systems that incorporate actual walking are likely to generate greater immersion and hence a greater sense of presence. However, this might come with several undesired side effects and a steeper learning curve. Kreimeier et al.’s [58] initial evaluation of locomotion hardware further showed that VR treadmills felt safer and more intuitive and that they generated more realistic speed experiences compared to the walking-in-place technique (without treadmill). However, treadmills were also more demanding in terms of the physical effort associated with them and the sense of tiredness they generated.

Differences in presence level may also appear between two similar locomotion technologies. Hooks et al. [46], who compared the Omni Virtuix with the Cyberith Virtualizer VR treadmills, found that users reported higher presence levels when using the Omni Virtuix, which they categorized as a bowl-based omnidirectional treadmill (i.e., the low-friction walking surfaces on which users walk has a bowl-like shape), compared to the Virtualizer, which is a flat-based treadmill. The higher levels of presence were achieved due to what seems to be the superior immersiveness properties of the bowl-based treadmill (the Omni Virtuix). These included better performance, ease of use and comfort. Kreimeier et al. [58], who compared the same treadmills, reported that the Omni Virtuix generated a greater sense of safety but was less intuitive and more tiring than was the Virtualizer.

Studies that utilize IVEs to investigate environmental preferences and impact on behaviors commonly share a similar methodological approach. They capitalize on the ability to control and manipulate environmental elements and stimuli to systematically compare between one and several different environmental conditions using either within- or between-subject research design [49,59]. The comparison may take a more holistic approach, where two or more completely different environments are presented to participants [60] (e.g., green forest vs. urban environment) or an element-oriented comparison in which one or more environmental elements are manipulated but the overall environment remains similar in all conditions [47,48] (e.g., urban environment with or without greenery; see, for example, pilot study below). The IVEs that are used in these studies can either be hypothetical ones that simulate a typical generic environment [47,48] or they can represent an existing place [50]. In the latter case, researchers may test how hypothetical or planned environmental changes may affect the behavior and perception of people by manipulating the IVE accordingly [50,61].

IVEs are also used as a complementary visualization technique that allows an immersive, dynamic and near-realistic representation [49]. Such representations allow researchers to present their participants vivid, more complex environments and situations that are superior in terms of sense of presence to more traditional representation techniques of textual description, static picture and even non-immersive virtual environments [62,63].

IVE technology makes it possible to integrate many useful data collection techniques about human behavior. While a comprehensive review of these techniques is beyond the scope of the article, we briefly introduce the main types of data that can be recorded in IVE to study human behavior while emphasizing methods relevant for investigating walking experience and momentary wellbeing.

*Self-reports*—Probably the most common method for recording participants’ personal experiences and behaviors, self-reports provide researchers with a simple way to record relevant introspective data about preferences, experiences and wellbeing. However, while there is much information on best practices of implementing and analyzing self-reports, the method is often criticized for being easily biased due to wording, order of questions and other factors related to the design of the questionnaire and characteristics of participants [64]. Researchers use self-reports in IVEs in the form of a simple questionnaire or as part of a stated preferences experiment in which participants rank, rate or choose their preferred option from a set of available alternatives [48,62,63]. Importantly, IVE studies facilitate a variety of options for triggering questionnaires based on location, time and other events that occur (e.g., when a change in participants’ behavior, such as their walking speed, is detected). This makes it possible to ask questions at very precise moments. Self-reporting can also take place before or after the IVE experiment episode using a more traditional questionnaire [47,49]; however, questions prompted in situ may make it possible to overcome typical recall biases, which is important in studies about momentary experiences and wellbeing.

*Internal sensors and system logs*—IVE hardware is often equipped with built-in sensors that support the technology’s basic operation. Such sensors make it possible, for example, to extract data about the orientation of the HMD headset. Furthermore, the system allows a rather straightforward logging of participants’ positions in the virtual environment, one of the most common data that researchers utilize [47,48]. More advanced headsets include eye-tracking sensors that can track pupils’ movement. Such eye-tracking capabilities can be invaluable in studies that investigate the relations between observed objects and behavioral outcomes, where it is important to control whether participants noticed an object and for how long. 

*External sensors*—It is possible to integrate various useful external sensors in IVE simulators that can further enhance the measurements of physiological and behavioral indicators. External sensors may include, for example, additional devices that measure body movement and posture. Biosensors, such as electrodermal activity (EDA) sensors used to record emotional arousal, cardiovascular activity sensors that are used as markers for both mental and physical states and even electroencephalography (EEG) that records the electrical activity of the brain [60,65] are another prominent example. Synchronization between the data of the built-in sensors and the external sensors can be done in a post hoc manner based on time fields. If a more straightforward synchronization is required, a full integration of external sensors with the VR system is usually possible through APIs and SDKs that are available with many commercial sensors.

*Qualitative techniques*—Analysis of human behavior in IVEs may also rely on qualitative methods, including interviews, observations and records of comments that participants make during IVE sessions [50]. Due to their nature, qualitative techniques are usually restricted to pilot studies and studies with a relatively small number of participants.

While IVEs offer many methodological opportunities and useful tools for evaluating walking experience, only a few studies have employed the technology. Those studies that did implement some locomotion interaction in IVEs were typically not about walking per se, but rather about related outputs, especially cognitive perception and memory of space and distance. Furthermore, these studies implemented inferior locomotion technologies, such as a joystick or hand-held gaming controller [66], or they only permitted participants to walk within the limits of room dimensions [67]. Our review shows that the research of locomotion and walking behavior in IVEs is still largely unexplored [68]. Consequently, there has been only little experience with the implementation of IVEs in studies about walking and especially about the usage of more advanced locomotion techniques that generate a strong sense of embodiment. In the following section, we pilot test the technology, after which we discuss and suggest guidelines for a methodological framework which could help promote this type of research.

## 3. Implementation of a VR Walking Simulator: A Pilot Test

To test and demonstrate the potential of IVE technology as a research tool for studying walking behavior and its impact on wellbeing, we employed a naïve experiment design in which participants were asked to take a short walking trip in a virtual urban environment. An HMD-based walking simulator that we developed for this purpose was used. The aim of this pilot test was to evaluate the capabilities and opportunities that the technology offers, and to test the feasibility of implementing a VR walking simulator for research purposes with human participants. In particular, we were interested in evaluating the technology’s ability to measure high-resolution parameters relevant to walking behavior and wellbeing, and their relation to the characteristics of the walking environment (e.g., the presence of greenery or traffic density). The study was approved by the institutional review board of Tel Aviv University (IRB no. 0000700-1).

### 3.1. Materials and Methods

#### 3.1.1. The VR Simulator

**Figure 1:**
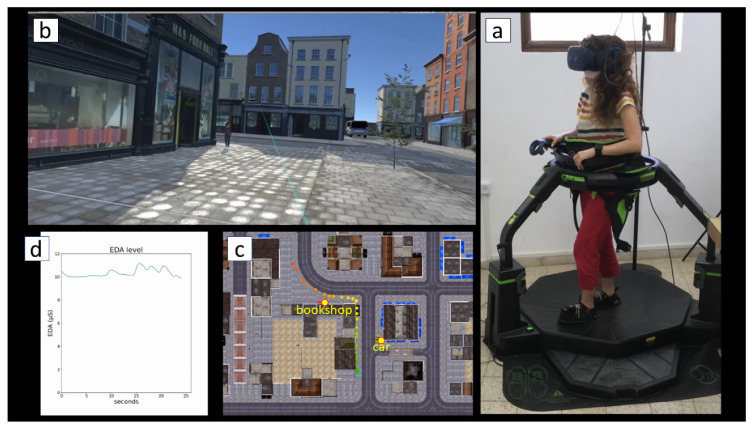
Participant 2 on her 2nd run, 25 s into the experiment. (**a**) The walking posture on the Virtuix Omni treadmill. (**b**) The immersive display. (**c**) The walking route (“GPS” points). The color of points represents time (green tones at beginning of the route and red ones toward the end of the route). (**d**) EDA level along the experiment (x axis—time in seconds since the beginning of the experiment; y axis—electrodermal activity (EDA) level in μS). See a dynamic representation of Figure 1 in Video S1.

The simulator that was developed is comprised of four main components:

1. A walking controller. We used a commercial Virtuix Omni treadmill unit, an omnidirectional treadmill that allows walking in virtual environments. Walking in the virtual environment is performed by sliding on the low-friction surface of the Virtuix Omni using designated (over)shoes while connected to a harness (see Figure 1a and Video S1). While the movement is relatively simple, it is not entirely identical to natural walking due to the need to slightly lean forward during the walk and the rather “mechanical” turns that the treadmill imposes (see Video S1).
2. A visual display unit. An HTC Vive Pro Eye HMD unit incorporating built-in eye-tracking capabilities was used. The HTC has a resolution display of 1440 × 1600 pixels per eye, refreshment speed of 90 Hz and a field of view of 110 degrees. The unit includes hand controllers that allow the user to interact with the virtual environment.
3. An IVE. The IVE was developed within Unity, a cross-platform game engine. In the current experiment, the development of the environment was based on a virtual template of a typical modern urban neighborhood of mix-used buildings—both residential and commercial—as well as roads and sidewalks. Additional virtual elements such as cars, trees and people were purchased in Unity’s Asset Store and added in order to enrich the environment.
4. The simulator software. The software that was developed by the authors served as the engine of the experiment. It facilitated setting the experimental conditions, controlling objects within the IVE (e.g., people and car movement), prompting questionnaires, logging data generated by the system (e.g., location coordinates) and more.

#### 3.1.2. Participants and Procedure

Four participants took part in the pilot test. Participants were volunteer colleagues, one male and three females (see personal characteristics in Table 1). Two of the participants had previous experience with the simulator; for the other two, this was the first encounter with a VR walking simulator.

**Figure 2:**
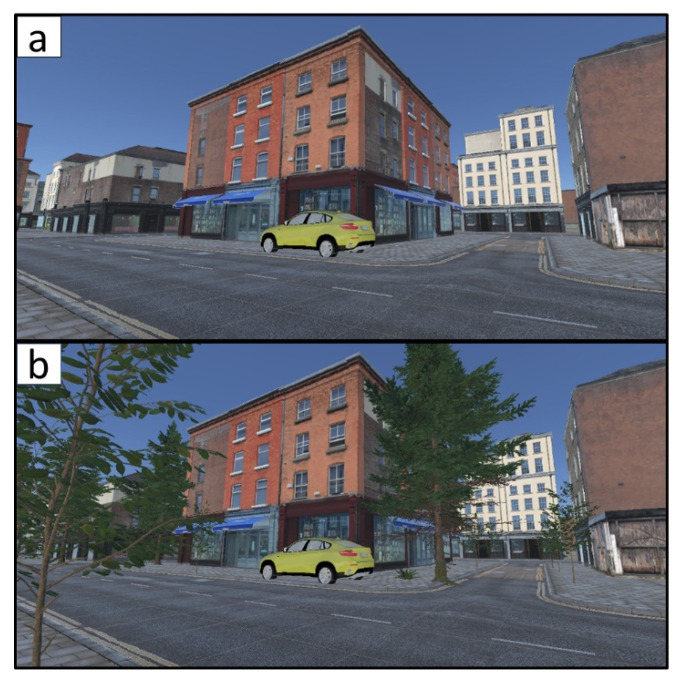
A 2-D view of the immersive virtual environment (IVE) extracted from near the starting point of the experiment. (**a**) control condition (no greenery); (**b**) green condition. The two environments are identical other than green elements that were used in the second environment. Pupil fixation on the yellow car that appears in the images was recorded using the VR headset’s eye-tracking sensor.

Participants were asked to walk two short routes of approximately 70 m each and report how enjoyable the walk was on a 7-point Likert scale at the end of each walk using their hand controller. The two routes were identical other than trees that were embedded along the sidewalk in the second route (see Figure 2). This design was intended to simulate a two-condition research design of (1) basic control scenario of a street without trees and (2) “green scenario” of the same street with trees. Upon arrival, participants were briefed on the task. The two novice participants were instructed on how to walk in the simulator and how to use the hand controller. Two types of external sensors—Empatica’s E4 wristband, measuring physiological data (e.g., EDA and cardiovascular activity), and two Gait Up Physilog 5 gait sensors (inertial units)—were then installed on the participants’ wrists and shoes, respectively. With the assistance of the experimenter, participants were asked to step into the Virtuix Omni treadmill and put on the VR headset. The experimenter inserted a participant ID number into the simulator software, initiated the experiment with the control condition and asked the participant to begin walking. After completing the first round, participants were asked to repeat the walk in the second (i.e., green) condition. Conditions were not randomized; all participants started with the control condition, which was followed by the green condition.

### 3.2. Results

Table 1 presents participants’ characteristics and the descriptive statistics of the variables that were measured for each of their walking runs. Variables are organized and presented in the table and in this subsection by the type of the data using the categories discussed in Section 2: self-reports, built-in sensors and system logs, and external sensors. Each result is discussed in its relevant methodological context while focusing on the implications of the data collection technique on studying walking, walkability and wellbeing in IVEs.

#### 3.2.1. Self-Reports

In order to test and demonstrate self-report options within IVEs, participants were asked to self-report their level of agreement with the statement “I enjoyed walking this route” (1—strongly disagree; 7—strongly agree). The question was prompted at the end of the route based on the position of participants (see Video S1) using a geofencing technique (i.e., the questionnaire was triggered when participants entered a predefined area).

Participants’ self-reports are presented in Table 1. All participants except for P3 found the green route (2nd run) more enjoyable. Participants reported an enjoyment level of 4.5 on average for the control condition and 5.75 for the green route. This difference was statistically significant (t = −2.611; *p*-value = 0.040; see Table 2). It should be noted that conditions were not randomized, which may have affected the results. For example, participants might have been more familiar with the task and technology after walking the first route, which may have improved their experience in the second run.

#### 3.2.2. Internal Sensors and System Logs

In the current simulator we utilized built-in sensors and system logs to track and record the spatial position of participants in the virtual environment during their walk (see Figure 1c and Video S1) in a similar way (though with greater accuracy) to GPS technology in the real world. Furthermore, we capitalized on the eye-tracking capabilities of the HTC Vive Pro Eye to record the duration of time that participants’ pupils were fixed on two selected objects: a yellow car that was parked at the beginning of the route and a bookshop display window (see Figure 1c for their positions; the yellow car is also shown in Figure 2). This technology made it possible to accurately associate between gaze durations on specific environmental stimuli and corresponding walking behaviors.

Some variation between participants in walk duration was observed (see Table 1). P3 completed the walk in the shortest time in both runs (25 and 23 s, respectively) while P4 was the slowest. P4 had an exceptionally slow walk since she did not adapt to the somewhat unnatural walking posture and mechanism that the simulator requires during the two short sessions. Since the route was identical in all runs, walking distances that were recorded were very similar, with the exception of the first walk of P4, who did not take the most direct path (79.45 m), probably due to her difficulty walking on the omnidirectional treadmill. Even with the small study sample, walking distance was significantly shorter at the 10% level in the second run (Table 2). This is likely due to the fact that participants were more familiar with the route on their second attempt. Some variation in speed between participants can be observed, ranging from 2.33 kmph (P4, first run) to 11.03 kmph (P3, second run). Walking speed was very high on average (7.99 kmph). However, the fast walking speeds that were recorded are a result of inadequate calibration of the VR simulator which was translated into inaccurate locomotion behavior. This emphasizes the importance of calibrating the VR treadmill (see more details below in the gait analysis).

Eye-tracking measurements revealed large variance between participants and between runs (see Table 1). All participants had their eyes fixed on the first target of the parked yellow car in all runs except for P3 in the second run. P4 observed the car for extremely short periods of 44 and 65 ms in the first and second runs, respectively. In contrast, the bookshop was observed in only five out of the eight runs, but for relatively long periods that ranged between 1116 and 8776 ms. It is possible that the bookshop display window was less noticeable due to its position away from the central vista of the street. However, once observed, it generated more fascination and consequently longer observation durations, which may have affected the walking experience.

#### 3.2.3. External Sensors

Two types of external sensors were used in the pilot test: (1) The E4 biosensor wristband that provides physiological data about EDA, cardiovascular activity (heart rate, interbeat intervals) and skin temperature. This information can be used for assessing the physical condition of the body as well as people’s mental and emotional states (e.g., stress level) [69]. (2) The Physilog 5 inertial unit that is used for gait analysis. Gait analysis is commonly used to detect and treat people with medical conditions, but studies have demonstrated that gait is also indicative of emotional state [70].

EDA levels (see Table 1) ranged between 4.435 μS (P4, first run) to 28.401 μS (P1, second run). As indicated in Table 2, participants had significantly higher EDA levels in the second run than in the first run (16.0 vs. 12.46 μS, *p*-value = 0.023). This is probably a result of increased sweating due to the walking activity, which also elevates EDA level, and is not necessarily an indication of increased emotional arousal. In contrast, standard deviation of EDA was lower, though not statistically significant, in the second run (see Table 2). This can be explained by the rapid increase in sweating at the beginning of the walking experiment that resulted in high variability in EDA levels, which was followed by stabilization of temperature and sweat in the second run.

Heart rates (HR) ranged between 82.084 (P4, first run) and 115.121 (P1, first run) beats per minute. It showed a very similar pattern to the EDA: there was an increase in HR from first run to second run and a stabilization of the beats in the second run, reflected in lower standard deviations. Standard deviation of HR measurements was statistically significant in the first run compared to the second run (5.20 vs. 1.77, respectively, *p*-value = 0.044). 

Gait sensor data was analyzed using the python’s sensormotion package. Converting the three-axis accelerometer and gyroscope information into meaningful gait parameters was not straightforward due to the unnatural walking posture and leg movement that the Virtuix Omni treadmill imposes. In practice, each participant adopted a slightly different walking strategy (e.g., some slid on the treadmill surface while keeping their feet connected to the surface, some leaned forward more than others). For that reason, script parameters had to be calibrated for each participant separately. Based on our experience in the pilot test, it seems that indicators that were extracted from the external gait sensors were not entirely reliable when implemented in the Virtuix Omni and they should be treated with caution. 

Gait analysis revealed inconsistency between the number of steps and route length. As the route was approximately 70 m long, it should have taken an adult participant approximately 90 steps to complete the walk. However, other than P4, who had difficulties adapting to the VR treadmill, participants required between 20 and 41 steps to complete the journey. This means that each step in the treadmill was translated to longer than it should have been in the virtual environment, which most likely affected the immersiveness qualities of the simulator and hence participants’ sense of presence. Gait sensors allow researchers to generate additional indicators that may be found useful in some studies. Step regularity and step symmetry are two such indicators (see Table 1 and Table 2). This type of marker is commonly used to detect medical conditions, but it might also turn out to be useful as a behavioral indicator or at least as a control variable in studies on walkability. Nevertheless, its implementation in a VR simulator should be further investigated and validated before it can be used.

## 4. Methodological Guidelines for IVE Study Design

Based on the pilot test, our experience in conducting various types of IVE experiments, and a review of the literature, we propose general guidelines for designing an IVE research experiment on mobility in general and on walking and walkability more specifically below. Our aim here is not to cover the technical details, as these are both too broad in scope and likely to change rapidly with the development of the technology. Instead, we focus on the major decisions that researchers face when designing a study which employs the technology and on essential procedures that should be considered. Useful technical guidelines for performing IVE experiments can be found elsewhere see for example [49].

### 4.1. Choice of Technology

Choosing the technology is a crucial decision that may affect many research projects conducted in the lab. Researchers should keep in mind that the implementation of IVE is resource-demanding and requires dedicated hardware, lab space, software and expensive programming hours in cases in which in-house programmers are not available [48,62,63]. Therefore, the choice of adequate technology and devices should account for basic technical factors, technological aspects and costs according to research objectives. It is crucial to define the study’s main focus. For example, the research may require the generation of a strong, realistic sense of walking. If this is not the case, a simpler and less expensive walk-in-place technique or joystick might be sufficient. In some studies, special data may be sought that rely on unique hardware. To compare devices and select the optimal one, further advice can be found through available studies [46,57,58], consultation with colleagues, manufacturer specifications, and technical support. The type of both internal and external sensors that are employed should also be carefully considered. Studies that investigate affective reaction to specific environmental events and stimuli may consider implementing eye-tracking technologies in combination with biosensors, for example. This constellation may allow high-resolution investigation of the stimuli’s influence.

### 4.2. IVE Design

As discussed in Section 2, perceiving a simulated urban environment as unrealistic or unnatural may have adverse consequences on a study’s results. Two further concerns may be that participants may differ in the extent to which the experimental environment seems realistic to them, or that the control and treatment environments may differ in perceived realism or induced sense of presence, which may act as an alternative explanation to the results. To attenuate these concerns, it is advisable to pretest the environments and stimuli to verify that the treatment and control do not significantly differ in their qualities and level of perceived realism. Several validated inventories may be used for this purpose such as the common multi-dimensional ITC - sense of presence inventory [71] as well as shorter scales such as the spatial presence experience scale [56]. In the pretest phase, these indicators can be used to test the quality of the environment and to validate the experimental task. In the analysis phase, the indicators may be used to filter unconvinced participants or be considered as moderators or covariates. The quality of the displayed environment should also be tested for exceptional adverse side effects that it may generate (e.g., by using the negative effect scale of the ITC sense of presence inventory). We recommend reporting presence scores of the IVEs and especially the dimension of spatial presence as a standard in IVE studies. This may help standardize the field. Researchers should keep in mind that while basic environments are relatively simple to generate (e.g., by relying on available virtual environments as done in the pilot test), highly realistic, interactive, and place-specific environments may be costly to produce. Further, to facilitate realism, a synchronization between actual body movement and sense of advancement in the virtual environment should be achieved. To this end, treadmills or other locomotion devices should be calibrated (as discussed above in Section 3.2.2 and Section 3.2.3).

### 4.3. Sampling

*Statistical power.* Our experience with past IVE experiments shows that IVE studies often result in a larger effect size, which can be twice as strong compared to more static, less vivid display techniques, such as still images. Still, since IVE studies involve resource-demanding procedures, they tend to rely on small sample sizes, which result in relatively low statistical power. This problem may worsen with increasing measurement errors and sample variation [72]. Thus, we advise conducting a power analysis or at least adopting some rules of thumb for the approximate sample size required to detect an effect. To develop intuition for effective sizes, it is advisable to refer to the results of the extant literature regarding the treatment of choice, rely on your own past studies if relevant, or extract estimates from a “dirty” pilot test.

Given the labor-intensive nature of data collection in VR studies, it may be worthwhile to consider additional ways to increase statistical power that do not involve increasing sample size. These might include decreasing the random error by using reliable and validated measures as well as relatively homogenous samples, strengthening the manipulation, if possible, to increase the between-group differences, and employing a within-subjects manipulation where possible.

*Conscious sampling choices and sample control variables*. Typical IVE experiments, as in the case of our pilot test above, often rely on a convenience sample. It is thus important to be conscious of selection factors by which the sample differs from the target population which may undermine generalizability. To start with, sampling from a population of “experts,” e.g., students of architecture or urban or environmental studies, may be not only non-representative but disadvantageous. Experts are more likely to be aware of the potential impacts that urban planning intervention and environmental cues may have on walking experiences. Their perception of the environment may differ in non-trivial ways from that of the average population, depending on their expertise, and this may manifest in their responses. For instance, their self-reports about the extent to which they enjoyed the experience or their appraisal of the environment’s level of realism may be informed by their a priori knowledge of the field and their professional criteria. 

Some personal characteristics relevant to this type of study should be collected and used as control variables and in some cases even considered as exclusion criterion, depending on the research goals. For instance, as found in our pilot test, level of experience with IVE technology may affect participants’ performance (see also [73]) and influence walking experience due to an “overwhelming effect” that first-time users encounter [48]. Therefore, familiarity with IVEs should be controlled, for instance by asking *“How many times in the past have you experienced a virtual environment using a VR headset? 1. Never before (this is my first time); 2. A few times (1–5 times); 3. Multiple times (between 5 and a few dozen times); 4. I often use virtual reality headsets.”* Other unique factors that should be documented may include vision disorders, which can affect the quality of display in some headsets or which may require more cautious calibration. Walking impairment and other medical conditions may also affect performance and can affect the outputs of the sensors used (e.g., gait and cardiovascular indicators).

### 4.4. Data Collection Procedure

Researchers should employ a well-defined data collection protocol to obtain high-quality data and reduce potential biases. Two main concerns in this regard are: (1) Participants are not familiar with the novel technology and hence their attention is occupied with operating the devices rather than with the experimental task. Such bias may manifest in a variety of indicators, such as walking duration and number of steps in the case of P4 in our pilot test (see Section 3 above). (2) The relatively complex data collection procedures that may be involved in such studies may affect data quality if procedures are not performed in a meticulous and standardized manner. This requires careful training of the research team. Researchers should also make sure they minimize potential discomfort for participants, especially those engaging in IVE for the first time. In this subsection, we emphasize several procedural tools that may help mitigate these potential problems.

*Training session.* Researchers need to ensure that participants have sufficient knowledge and experience to operate the hardware, navigate through the IVE, and use other controllers if necessary. Therefore, a training session will usually need to take place before the experimental IVE task begins. When complex tasks or usage of technologies are involved, the training session can start with an explanatory video that demonstrates the usage of the devices, followed by a training session. Researchers should confirm that at the end of the training, participants are capable of using the system satisfactorily.

*Instructions for participants.* Participants should receive a clear, accessible and standardized explanation which justifies the use of the equipment without revealing the experimental manipulation. Thus, a complete experimenter’s protocol should be prepared for the experiment, including instructions for all stages of contact with participants. The research team should be trained in advance in using the standardized instructions and in helping respondents with the equipment while respecting their privacy and minimizing direct physical contact. Furthermore, participants should be informed about potential risks involved in using IVE—such as fatigue and nausea—when signing informed consent, and they should receive full debriefing at the end of the experiment. These measures should be incorporated in the ethical application.

*A well-formulated experimental protocol and training of the on-ground research team.* It is crucial, especially when a complex data collection procedure is involved, such as when using a combination of treadmill and external sensors, that research assistants know the exact order of the research stages and practice them in advance. It is also important that the team is trained in handling basic technical malfunctions. This may help avoiding loss of participants, which can often be costly in this type of study.

*IVE session duration.* Kourtesis et al. [74] found that VR sessions should not be longer than 55 to 70 min when IVEs are of high standard and participants are familiarized with the system. However, their study did not incorporate locomotion hardware with novice users. Based on our experience, we recommend that sessions should be substantially shorter, preferably no longer than 10–15 min, though this should be further examined more systematically. While this highly limits the total number of scenarios that each participant can be exposed to, it ensures that adverse side effects associated with VR sickness will be reduced to a minimum.

### 4.5. Control Measurements

A few additional measurements should be considered in IVE studies that evaluate the effect of the environment on walking behavior and wellbeing. These are particularly important in studies that involve locomotion activity which generate more complex interactions with the displayed environment, as in the case of the pilot test above.

*Manipulation check: Attention to cues.* A basic type of manipulation check examines whether the participant ever noticed the experimental treatment. This is important in studies in which environmental differences between conditions are subtle, or respondents may differ in their motivation to participate. Traditional, self-reported indicators of attention, by which respondents are asked whether they have seen specific objects during their tour, are vastly unreliable, as noticing a cue with or without awareness does not dictate later recollection of the cue. Instead, it may be more indicative to track a respondent’s gaze using eye-tracking technology, as demonstrated in the pilot test. If unavailable, post hoc analysis of the participants’ field of view could be considered as a proxy.

*Measuring awareness.* To account for the demand effect (i.e., bias stemming from participants changing their responses based on their speculation about the study goal, aiming to assist or disrupt the experimenter), a post-experiment questionnaire should examine respondents’ awareness of the manipulation. This can be done in several ways. First, respondents could be asked, on a 1–7 scale, *“To what extent did you feel you knew what the researchers were investigating in this research?”* [75]. Another option is to ask an open question such as *“What do you think the study was about?”* It is often the case that participants evidencing a genuine understanding of the link between the manipulation (e.g., experiencing greenery in the environment) and outcome variable (e.g., wellbeing) should be excluded from the analysis, as they may be prone to counteract the effect [76]. Note that what matters is not lack of awareness about the manipulated factor—that is, having noticed the greenery—but instead, lack of awareness of the expected influence of that manipulation.

### 4.6. Preregistration

As demonstrated in the pilot test study, IVE studies offer the extraction of a particularly rich array of information including reaction times, gaze data, biosensor information, and more. Conducting multiple comparisons and analyses severely undermines confidence in the results. We therefore recommend pre-registering data collection procedures, analysis protocol, expected results and hypotheses, and inclusion criterion up front using public repositories and services like Open Science Framework and AsPredicted [77]. This practice supports the confidence in the result and may help advance the field.

## 5. Conclusions

To date, the utilization of IVE with locomotion technology is limited. The current article presents the opportunities that the technology offers alongside its limitations. Furthermore, it provides several guidelines for implementation which could help standardize the field in the future and give some first directions to novice researchers. IVEs offer few distinct advantages—most notably, the experimental research design that IVEs support improves causality inference while maintaining high ecological validity [48]. As demonstrated in the pilot test, IVE technology allows the collection of advanced high-resolution information on human behavior using various sensors and sophisticated self-report, which is hard to impossible to obtain under real-world conditions. These technological capabilities may assist researchers in associating between specific environmental stimuli and psychophysiological and behavioral reactions, and hence wellbeing.

However, IVE technology is still not a fully mature technology and carries several limitations. First, generating high-fidelity IVE simulation is still a challenging task. Immersiveness problems that may reduce the sense of presence include: (1) limited realism of humanoids’ and cars’ behavior and movement; (2) a limited level of interaction between users and IVE; (3) the walking mechanisms in treadmills, which are still imperfect; and (4) the resolution of the display and field of view (in HMD) which are still relatively low. It is thus advisable to incorporate indicators which directly examine the consequences of such limitations, as we detail in Section 4. Second, the implementation of IVEs requires substantially more resources compared to more traditional surveys and experimental methods [48,62,63]. Third, the use of IVEs in general and of walking simulators in particular still requires some professional knowledge which is likely to be a barrier for implementation. Fourth, as explained in Section 4, an IVE session is a rather long procedure, which may limit the number of participants that can be drafted. Furthermore, the number of trials that each participant may experience is also limited since it is advisable to restrict the durations of IVE sessions. Finally, and probably most crucial, is that IVEs make it possible to answer very specific types of questions on walking and wellbeing, namely, how momentary exposure to environmental elements may affect walking behavior and/or wellbeing. Moreover, studies in IVEs are often limited to investigations of participants in novel environments. Importantly, many of the limitations mentioned above are likely to be resolved with the advance of the technology and its continued adaptation to research purposes.

Since IVE technology is still in its infancy, additional research is required to further standardize the tools and procedures that are to be used. In this regard, it is important to investigate the quality of various technologies and compare different models and manufacturers of IVE simulators. Beyond methodological aspects, future research should critically test the accumulated findings from past studies that utilized more traditional methods and evaluate the impact of prominent environmental factors on walking (see review in the introductory section). Oftentimes studies report inconsistent and even contradictory findings about the impact of specific environmental factors. IVEs make it possible to test these factors systematically using experimental designs.

Extracting objective indicators about mood, mental and physical states and cognition based on mobile brain/body imaging, such as EEG [78], biosensors [69], human body thermal radiation sensors [79,80] and other available technologies constitute another promising field of investigation. The implementation of some of these technologies in real outdoor environments was already supported and tested in the past [69]. However, real environments pose challenges for interpreting results due to their greater complexity, which can be at least partly avoided in indoor IVE experiments. Another promising research approach that should be considered involves implementing qualitative and mixed methods to investigate walking experience. Researchers could integrate, for example, insights about momentary experiences that are collected in IVEs with knowledge about daily routines that are extracted from interviews and observational methods. Similar approaches have been tested in real-world environments [40,81] and their implementation in IVEs studies could constitute a fruitful extension of the research that can help bridge the gap between studies focusing on momentary wellbeing and those focusing on longer-term wellbeing. Finally, the study of walking in IVEs may support clinical and public health works. The method can combine between free-living observational designs and controlled laboratory studies, with or without intervention [82], and help underlying the clinical effectiveness of walking, particularly under challenges in the retrieval of standardized free-living walking patterns and self-reported well-being scales.

## Figures and Tables

**Table 1:** Participants’ characteristics and per-run descriptive statistics of recorded variables.

	P1		P2		P3		P4	
Participants’ Characteristics								
Gender, Age Group	Male, 30–45		Female, 30–45		Female, 30–45		Female, 30–45	
Experience with Simulator	Experienced		Experienced		Novice		Novice	
	1st Run	2nd Run	1st Run	2nd Run	1st Run	2nd Run	1st Run	2nd Run
**Self-reports**								
*Real-time questions*								
I enjoyed walking ^a^	5	6	4	6	5	5	4	6
**Built-in sensors**								
*Location and time*								
Walking duration (seconds)	30	31	30	26	25	23	123	59
Walking distance (meters)	74.49	71.65	71.05	71.87	71.76	70.50	79.45	73.78
Speed (kmph)	8.94	8.32	8.53	9.95	10.33	11.03	2.33	4.50
*Eye tracking ^b^*								
Yellow car (ms)	1110	1021	1606	3401	910	0	44	65
Bookshop (ms)	2031	1116	0	0	0	0	8776	0
**External sensors**								
*Biosensors*								
EDA (avg μS, stdv)	23.055, 3.260	28.401, 0.600	9.745, 0.413	10.320, 0.331	12.596, 1.182	15.990, 0.339	4.435, 0.611	9.302, 0.458
HR (avg bpm, stdv)	115.121, 3.555	100.473, 2.241	83.245, 1.705	92.255, 0.256	102.234, 4.215	110.728, 0.474	82.084, 11.335	86.249, 4.127
*Gait (inertial) sensors*								
Number of steps	32	26	41	36	24	20	108	55
Cadence (steps/min)	70.42	58.76	71.68	77.14	74.93	59.08	56.64	57.73
Step regularity	0.356	0.272	0.093	0.132	0.139	0.225	0.162	0.167
Step symmetry	0.925	0.902	0.601	0.841	0.622	0.806	0.844	0.895

kmph—km per hour; ms—milliseconds; EDA—electrodermal activity; HR—heart rate; μS—microsiemens; bpm—beats per minute; avg—average; stdv—standard deviation. ^a^ Level of agreement with the statement “I enjoyed walking this route” (1—strongly disagree; 7—strongly agree). ^b^ Total duration in milliseconds of pupils’ fixation on specified elements.

**Table 2:** Comparison of average values and t-tests between experiment conditions.

	Basic Condition (1st Run)		Green Condition (2nd Run)		T-Test (Paired, 1tail)	
	Average	Stdv	Average	Stdv	t-Statistics	*p*-Value
**Self-reports**						
I enjoyed walking	4.50	0.58	5.75	0.50	−2.611	0.040 **
**Internal sensors**						
Walking duration (seconds)	52.00	47.39	34.75	16.50	1.105	0.175
Walking distance (meters)	74.19	3.81	71.95	1.36	1.636	0.100 *
Speed (kmph)	7.53	3.55	8.45	2.86	−1.545	0.110
Yellow car (ms)	917.50	651.70	1121.75	1589.57	−0.359	0.372
Bookshop (ms)	2701.75	4161.14	279.00	558.00	1.138	0.169
**External sensors**						
EDA (avg)	12.46	7.83	16.00	8.77	−3.302	0.023 **
EDA (stdv)	1.37	1.30	0.43	0.13	1.557	0.109
HR (avg)	95.67	15.92	97.43	10.61	−0.315	0.387
HR (stdv)	5.20	4.22	1.77	1.80	2.488	0.044 **
Number of steps	51.25	38.47	34.25	15.33	1.416	0.126
Cadence (steps/min)	68.42	8.08	63.18	9.33	1.034	0.189
Step regularity	0.19	0.12	0.20	0.06	−0.320	0.385
Step symmetry	0.75	0.16	0.86	0.05	−1.877	0.079 *

See abbreviations and variable meanings in Table 1. * *p*-value < 0.1, ** *p*-value < 0.05.

## Data Availability

The data presented in this study are available on request from the corresponding author.

## Supplementary Materials

The following are available online at https://www.mdpi.com/1660-4601/18/2/364/s1, Video S1: An IVE Experiment that Incorporates VR Walking Simulator and Biosensors Measurements.
